# Systematically Studying the Optimal Amino Acid Distribution Patterns of the Amphiphilic Structure by Using the Ultrashort Amphiphiles

**DOI:** 10.3389/fmicb.2020.569118

**Published:** 2020-11-25

**Authors:** Shiqi He, Zhanyi Yang, Weikang Yu, Jiawei Li, Zhongyu Li, Jiajun Wang, Anshan Shan

**Affiliations:** Institute of Animal Nutrition, Northeast Agricultural University, Harbin, China

**Keywords:** antimicrobial peptides, amphipathicity, structure-function relationships, antibacterial mechanism, ultrashort amphiphiles

## Abstract

Amphipathicity has traditionally been considered to be essential for the de novo design or systematic optimization of antimicrobial peptides (AMPs). However, the current research methods to study the relationship between amphiphilicity and antimicrobial activity are inappropriate, because the key parameters (hydrophobicity, positive charge, etc.) and secondary structure of AMPs are changed. To systematically and accurately study the effects of amphiphilicity on antimicrobial properties of AMPs, we designed parallel series of AMPs with a different order of amino acids in a sequence composed only of Arg and either Trp (WR series) or Leu (LR series), under conditions in which other vital parameters were fixed. Furthermore, based on the WR and LR peptides that can form stable amphiphilic β-sheet structures in the anionic membrane-mimetic environment, we found that high β-sheet amphipathic was accompanied by strong antimicrobial activity. Of such peptides, W5 ([RW]_4_W) and L5 ([RL]_4_L) with a nicely amphipathic β-sheet structure possessed the optimal therapeutic index. W5 and L5 also exhibited high stability *in vitro* and a potent membrane-disruptive mechanism. These results suggest that the alternate arrangement of hydrophobic and hydrophilic residues to form a stable amphipathic β-sheet structure is an essential factor that significantly affects the antimicrobial properties.

## Introduction

Antibiotic resistance is one of the most severe issues currently facing our society (Wright, 2010). Under the conditions of antibiotic abuse, bacteria have developed multiple mechanisms to survive (Stewart and Costerton, 2001; Alexiou et al., 2017). The frequency of drug-resistant infections in patients has consistently risen, which has a severe impact on global public health (Qiao et al., 2018). The severe threat of drug resistance indicates that conventional antimicrobials are lagging behind the evolution of microbes (Reardon, 2014; Hancock, 2015). Therefore, the development of new antimicrobials as a promising strategy to combat continual mutant bacteria is urgently needed.

As a critical defensive line for organism immune systems, antimicrobial peptides (AMPs) have been the research hotspot (Dong et al., 2019). Compared to conventional antimicrobial agents, AMPs possess effective and broad-spectrum antimicrobial activity, faster sterilization efficiency and lower resistance due to a unique physical sterilization mechanism (Sato and Feix, 2006; Moghaddam et al., 2015). Generally, most AMPs exhibit amphipathic structures, which share the common characteristic of being cationic, hydrophobic and amphiphilic (Li et al., 2016). Among them, amphiphilicity is characterized by the clustering of amino acid residues in a dispersed or completely separated manner to form hydrophobic and cationic surfaces (Nikken et al., 2013), which is a significant structural feature of AMPs. Some studies have suggested that amphiphilicity plays an essential role in the antimicrobial properties of AMPs, which facilitates interactions with anionic surfaces and insertion into the microbial membrane, causing direct membrane lysis (Kim and Cha, 2010). However, this powerful lytic killing process usually comes at the cost of substantial side toxicity to healthy cells, such as erythrocytes, which is considered to be one of the main bottlenecks of the therapeutic window for application. Typically, perfect amphiphilic distribution leads to great broad-spectrum antimicrobial activity, simultaneously accompanying increased toxicity against healthy cells (Blondelle and Houghten, 1992; Hollmann et al., 2016; Khara et al., 2017). Of note, many recent studies have indicated that the imperfect amphiphilic distribution appears to be related to high antimicrobial activity and weakened cytotoxicity (Hawrani et al., 2008; Kim and Cha, 2010; Mihajlovic and Lazaridis, 2012; Zhu et al., 2014; Juretić and Simunić, 2019; Chen et al., 2020). Hence, AMP design has evolved from early perfect amphipathicity to imperfect amphipathicity.

Early strategies that investigated the impact of imperfect amphipathicity replaced residues within the hydrophobic face with polar amino acids or substituted basic amino acids with hydrophobic residues along the cationic amphipathic region (Zhu et al., 2014; Chen et al., 2020). However, the above research methods were inappropriate because modulation of amino acid residues commonly induced simultaneous changes in other vital parameters, such as hydrophobicity and positive charges. To settle these limitations, Wang and coworkers only permitted the alteration of amphipathicity via different order of amino acids in the sequence, while the others remained mostly constant (Liu et al., 2016; Wang et al., 2018). Whereas, this research method still has some shortcomings. For one thing, it has been quite difficult to elucidate the relationship between amphiphilicity and antimicrobial properties of AMPs by a single amino acid sequence alone. For another, changing the amino acid sequence may result in the transformation of the secondary structure of AMPs. However, it is meaningful to study the relationship between amphiphilicity and antimicrobial activity of AMPs only when a peptide can form a stable amphiphilic structure.

Therefore, to study the effect of the change of primary structure (amino acid order) on the secondary structure of AMPs and further investigate the relationship between amphipathicity and antimicrobial activity of AMPs with stable secondary structure, we first designed peptides with a different order of amino acids in the sequence under conditions in which critical parameters were fixed. Next, AMPs with the stable secondary structure were screened out by circular dichroism further to study the relationship between amphiphilicity and antimicrobial activity.

To fully study and eliminate the effects of amino acid composition on the properties of peptides, Trp and Leu were chosen to represent aromatic and aliphatic hydrophobic residues, respectively. Moreover, Arg was selected to represent the hydrophilic amino acids because it was charged under physiological conditions (Strøm et al., 2003), and the side chain guanidinium groups of Arg can form strong H-bonds with the phospho-rich membrane surface of the bacteria, enhancing antimicrobial potency (Zou et al., 2007; Veiga et al., 2012; Yang et al., 2019). Moreover, the appropriate cationic residue is an essential condition for electrostatic attraction between cationic AMPs and anionic microbial membranes (Wen et al., 2007; Lum et al., 2015). The results of Wang et al. found that six cationic residues were essential for the AMPs to enhance antifungal activity. Still, four cationic residues were sufficient for the antimicrobial activity of the most AMPs, suggesting that bacteria were less sensitive to a highly cationic peptide than fungi due to the thick fungal cell wall (Wang et al., 2018). In this study, to investigate the effect of AMPs on bacteria, four cationic residues (+5 net charges) were used. According to the above process, we designed two series of AMPs differing only in the use of Trp (WR series) or Leu (LR series) paired with Arg, based on parent peptide 1 (RLRLLLRLR-NH_2_) and peptide 7 (RWRWWWRWR-NH_2_) (Wang et al., 2018).

To consider the change in the secondary structure of peptides, we assumed that the peptides exhibited either α-helix or β-sheet structure and calculated hydrophobic moments, respectively (Table 1). Finally, the C-termini of the WR and LR series were aminated to enhance antimicrobial activity and improve stabilization.

**TABLE 1:** WR and LR peptides’ design and their key physicochemical parameters.

**Analogs**	**Peptide**	**Sequence**	**Theoretical MW^*a*^**	**Measured MW**	**Net charge**	**H^*b*^**	**μ *_*H*_*^*c*^**	
							**α helix^*d*^**	**β sheet^*e*^**
WR	W1	WWRWWRRRW–NH2	1573.81	1572.85	5	0.801	0.890	0.250
	W2	WWRWRRRWW-NH2	1573.81	1572.85	5	0.801	0.793	0.474
	W3	WRRWRWRWW-NH2	1573.81	1572.85	5	0.801	0.560	0.474
	W4	WWWRRRRWW-NH2	1573.81	1572.85	5	0.801	0.448	0.250
	W5	RWRWRWRWW-NH2	1573.81	1572.85	5	0.801	0.435	1.199
	W6	WRRRWWWWR-NH2	1573.81	1572.85	5	0.801	0.415	0.250
	W7	RRWWWWRRW-NH2	1573.81	1572.85	5	0.801	0.358	0.250
	W8	RRWWWRWRW-NH2	1573.81	1572.85	5	0.801	0.279	0.974
	W9	RWRWRRWWW-NH2	1573.81	1572.85	5	0.801	0.264	0.474
	W10	RWWWWRRWR-NH2	1573.81	1572.85	5	0.801	0.291	0.474
	W11	WRRRRWWWW-NH2	1573.81	1572.85	5	0.801	0.203	0.250
	W12	WRRWRWWRW-NH2	1573.81	1572.85	5	0.801	0.137	0.250
LR	L1	LLRLLRRRL-NH2	1208.55	1208.56	5	0.496	0.715	0.189
	L2	LLRLRRRLL-NH2	1208.55	1208.56	5	0.496	0.637	0.413
	L3	LRRLRLRLL-NH2	1208.55	1208.56	5	0.496	0.445	0.413
	L4	LLLRRRRLL-NH2	1208.55	1208.56	5	0.496	0.349	0.189
	L5	RLRLRLRLL-NH2	1208.55	1208.56	5	0.496	0.341	1.016
	L6	LRRRLLLLR-NH2	1208.55	1208.56	5	0.496	0.329	0.189
	L7	RRLLLLRRL-NH2	1208.55	1208.56	5	0.496	0.297	0.189
	L8	RRLLLRLRL-NH2	1208.55	1208.56	5	0.496	0.255	0.791
	L9	RLRLRRLLL-NH2	1208.55	1208.56	5	0.496	0.212	0.413
	L10	RLLLLRRLR-NH2	1208.55	1208.56	5	0.496	0.228	0.413
	L11	LRRRRLLLL-NH2	1208.55	1208.56	5	0.496	0.146	0.189
	L12	LRRLRLLRL-NH2	1208.55	1208.56	5	0.496	0.126	0.189

**^*a*^Molecular weight (MW) was confirmed by mass spectroscopy (MS). ^*b*^Hydrophobicity (H) values means the total hydrophobicity (sum of all residue hydrophobicity indices) divided by the number of residues, and they were calculated from http://heliquest.ipmc.cnrs.fr/cgi-bin/ComputParams.py. ^*c*^Relative hydrophobic moment (μ_*H*_) values were employed to analyze the level of amphipathicity of all peptides. ^*d*^μ_*H*_ was calculated by using a consensus hydrophobicity scale and a side chain offset angle of 100°, which assumes that the peptides adopt an α-helical conformation (Eisenberg, 2003). ^*e*^μ_*H*_ was calculated by using a consensus hydrophobicity scale and a side chain offset angle of 180°, which assumes that the peptides adopt a β-sheet conformation (Eisenberg, 2003).**

The secondary conformation of the WR and LR peptides was first measured by circular dichroism (CD). Then, the biocompatibility of these peptides was evaluated. Next, the antimicrobial activities of the AMPs against various pathogens with or without physiological salts were determined. Finally, fluorescence spectroscopy and electron microscopy were used to study the antimicrobial mechanism.

## Results and Discussion

### Characterization of the Peptides

The molecular masses and purities of the peptides were verified by matrix-assisted laser desorption/ionization time of flight mass spectrometry (MALDI-TOF MS) and high-performance liquid chromatography (HPLC) experimental analysis. The measured molecular weight of each peptide was almost the same as the theoretical molecular weight, and the purity of all the peptides was over 95%, indicating that the syntheses were successful. Due to the same amino acid composition, each series of AMPs possessed their common features, including molecular weight, peptide length, net charge, and general hydrophobicity. Still, two series produced differences in the hydrophobic moment (α-helix or β-sheet). The detailed characterization of the WR and LR series are displayed in Table 1.

### Secondary Structure Analysis of the Peptides

To investigate the effect of different amino acid order on the secondary structure of peptides, a highly sensitive CD spectroscopy was used to monitor the conformational changes of WR and LR peptides in various environments. Typically, small unilamellar vesicles (SUVs) have been employed to investigate the binding of peptides with lipids (Wieprecht et al., 2000; Nichols et al., 2013). In this study, we are employing 1.5 mM 3:1 POPC/POPG SUVs to investigate the binding interactions that occur between these AMPs and anionic liposomes. Also, because micelles do not form bilayer membranes, peptides cannot be inserted entirely into micelles to form pores as is the case with phospholipids (Russell et al., 2012). Hence, as a simple model for anionic lipids of the microbial membrane, 30 mM sodium dodecyl sulfate (SDS) micelles (Watson et al., 2001) will also be used to isolate and investigate the surface binding interaction of peptides from the aggregation and pore-forming interactions that occur on binding to lipids.

**FIGURE 1:**
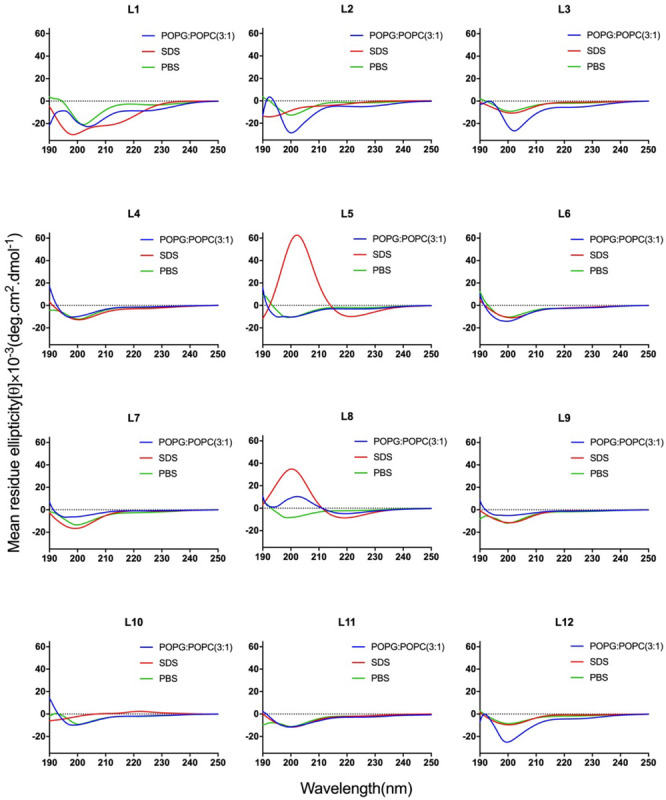
CD spectra of the LR peptides. Peptides were dissolved in 10 mM sodium phosphate buffer (pH 7.4) (green), 30 mM SDS (red), and 1.5 mM POPC: POPG (3:1) (blue). The mean residue ellipticity was plotted against wavelength. The values from three scans were averaged per sample, and the peptide concentrations were fixed at 150 mM.

**FIGURE 2:**
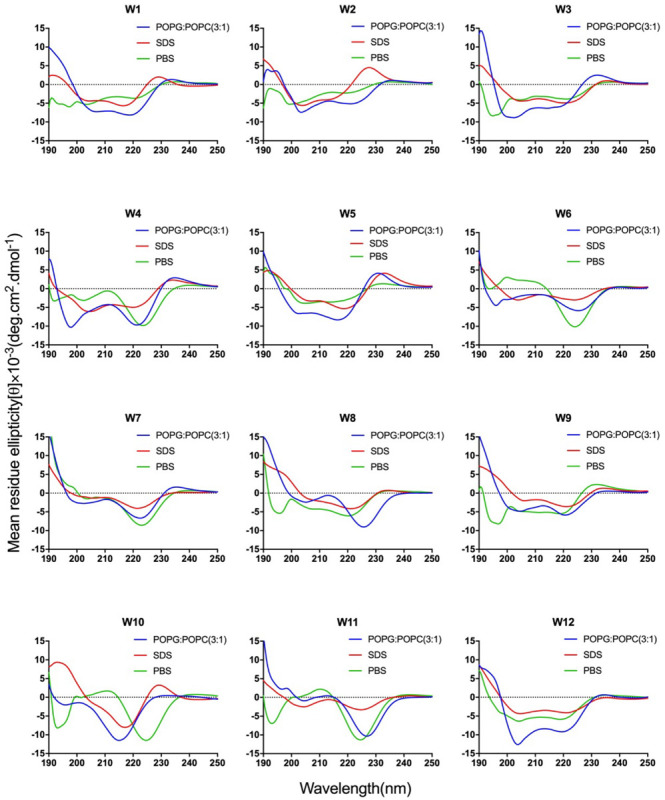
CD spectra of the WR peptides. Peptides were dissolved in 10 mM sodium phosphate buffer (pH 7.4) (green), 30 mM SDS (red), and 1.5 mM POPC: POPG (3:1) (blue). The mean residue ellipticity was plotted against wavelength. The values from three scans were averaged per sample, and the peptide concentrations were fixed at 150 mM.

**FIGURE 3:**
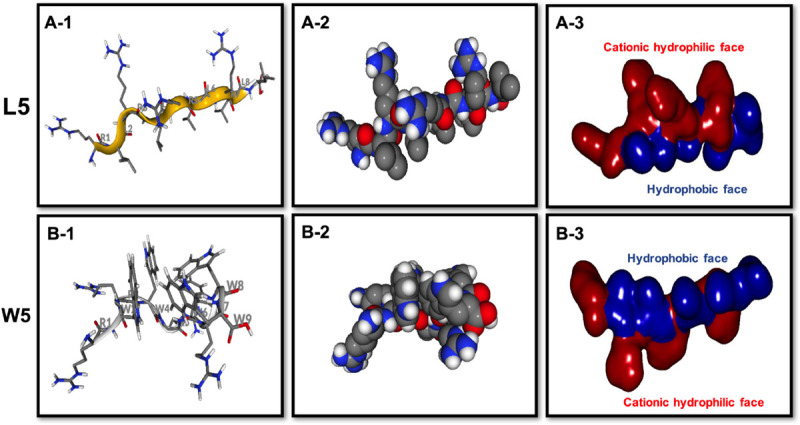
Three-dimensional structure projections of the basic amphiphilic framework of L5 **(A)** and W5 **(B)**. By default, the setting presents the β-sheet as yellow, α-helix as red, and extends structure as gray **(A-1,B-1)**. The space-filling models of L5 **(A-2)** and W5 **(B-2)** is shown in the middle panel. The electrostatic potential surface plot of L5 **(A-3)** and W5 **(B-3)** is in the right panel and the setting presents the cationic hydrophilic face as red and hydrophobic face as blue. The three-dimensional structure projection was predicted online with PepFold (https://bioserv.rpbs.univ-paris-diderot.fr/services/PEP-FOLD/).

CD spectroscopy of all peptides in 10 mM sodium phosphate buffer (pH 7.4) (PBS), and 30 mM SDS micelles and 1.5 mM 3:1 POPC/POPG SUVs was performed, and the results were shown in Figure 1 (LR series) and Figure 2 (WR series). In a sodium phosphate buffer, the CD spectra of all LR peptides showed negative band around 200 nm, which was characteristic of small unfolded peptides. According to the spectral characteristic of the LR peptides in 30 mM SDS micelles, only L5 and L8 exhibited maximum dichroic band around 200 nm and a minimum dichroic band at 216-220 nm, indicating a representative β-sheet conformation, other LR peptides adopted randomly unfolded conformations based on showing a negative band around 200 nm. Unexpectedly, in 1.5 mM POPC/POPG (3:1) SUVs, only L8 maintains the β-sheet structure, while L5 ([RL]_4_L) exhibited randomly unordered conformation. Unsimilar to the structure of L5 ([RL]_4_L) in lipid, the β-sheeted structure was established in the presence of lipids for [(KL)_*n*_K] peptides (Degrado and Lear, 1985; Castano et al., 2000; Béven et al., 2003). It should be noted that CD spectra of L5 observed in the presence of 3:1 POPC/POPG SUVs and SDS micelles were dissimilar indicating that L5 may interact with SUVs via more complex interactions than just surface binding (Russell et al., 2012). Hence, to observe the secondary structure of L5 intuitively, the PepFold was used to obtain the prediction. The measurement of L5 made by CD spectroscopy in SDS micelles is consistent with the structure predictions obtained using PepFold. As shown in Figure 3A, the predicted structure of L5 is characteristic of amphiphilic β-sheet structure, with leucine forming the hydrophobic surface and arginine constituting the cationic hydrophilic surface.

The CD spectra of WR peptides in 10 mM PBS, 30 mM SDS and 1.5 mM POPC/POPG SUVs are very similar in shape, showing a positive maximum ellipticity around 190 nm and two maximum negative ellipticities around 200 and 220 nm. Furthermore, due to the indole side chain of tryptophan, the CD spectra of several WR peptides show a positive band at 225–230 nm. This observation is in line with the CD spectrum of (RW)_4_ and tryptophan-rich peptide (HHC-36, KRWWKWWRR) in the presence of POPC/POPG vesicles (Liu et al., 2007; Nichols et al., 2013). Phambu et al. (2017) found that FTIR spectrum of (RW)_4_-NH_2_ consists of a mixture of β-sheet and β-turns structures in the presence of *E. coli* membranes. Moreover, Nichols et al. (2013) have identified that in tryptophan-rich peptides, exciton coupling derived from the close Trp-Trp interactions led to the formation of turn conformations. Hence, many WR peptides mainly showed β-sheet with certain turn conformation. Taking W5 as an example, the measurements of W5 made by CD spectroscopy in membrane-mimetic environments is different from the structure predictions obtained using PepFold. However, the predicted structure of W5 has obvious amphiphilic distribution, with tryptophan forming the hydrophobic surface and arginine constituting the cationic hydrophilic surface (Figure 3B).

Overall, the change of amino acid sequence has a significant effect on the structure of LR peptides, but has little impact on the structure of WR peptides. According to CD spectrum and calculated hydrophobic moment (α-helix or β-sheet), it would be evident that L8 and L5 ([RL]_4_L) interacted with surface binding can form amphipathic β-sheets, and also many peptides may have a higher tendency to form amphipathic β-sheets than α-helices.

### Biocompatibility Assays

AMPs express antimicrobial activity via a non-receptor-mediated membrane permeation mechanism, which possibly impacts the biocompatibility of AMPs to eukaryotes. Excellent biocompatibility is a prerequisite for AMPs to break through clinical application constraints.

**FIGURE 4:**
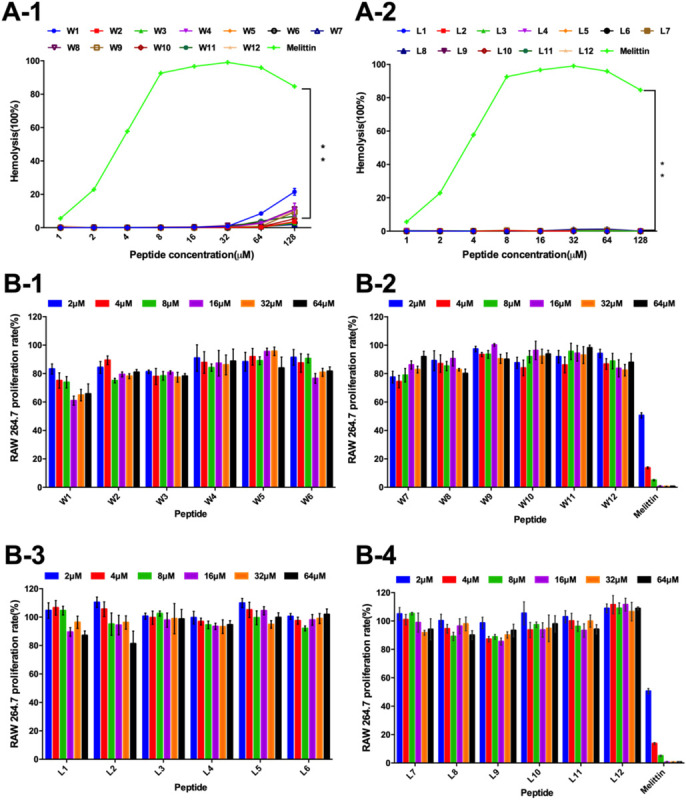
**(A)** Hemolytic activity of the WR **(A-1)** and LR peptides **(A-2)** against hRBCs. **(B)** Cytotoxicity of the WR **(B-1,B-2)** and LR peptides **(B-3,B-4)** in RAW 264.7 cells. The graphs were derived from an average of three independent trials.

Thus, the hemolytic activity of the two series of peptides was first assessed, and the hemolytic reaction is shown in Figure 4A. Compared with melittin, the WR and LR series both had notably lower hemolytic activity (*P* < 0.01). It was satisfactory that the LR series produced negligible hemolysis, less than 3% at all concentrations. However, compared with the LR series, the hemolytic activity of the WR series was slightly higher. Still, the hemolytic activity of most of the WR series was dose-dependent and remained below 10% at all concentrations. W1 and W4, however, were more hemolytic (21 and 11% hemolysis at 128 μM) than other WR peptides.

Furthermore, the cytotoxicity of the WR and LR series against RAW 264.7 macrophage cells was determined (Figure 4B). In accordance with negligible hemolysis, the LR series preserved an approximately 80-110% cell survival rate at all concentrations tested, demonstrating that majority of these peptides had inappreciable toxicity and excellent biocompatibility. Except for W1 and melittin, the cell viabilities induced by the WR series were above approximately 80%. Moreover, different concentrations of peptides had slight effects on cell viability.

Thus, all LR and most WR series peptides caused negligible hemolysis (below 10%) and maintained high cell viability (above 80%), further confirming that various order of amino acids in the sequence produced almost no hemolysis or cytotoxicity. However, the biocompatibility of the WR series was still slightly weaker than that of the LR series. This observation is consistent with the proposal of STRØM and coworkers that detectable hemolytic activity was observed for the peptides containing four or more aromatic residues, especially tryptophan (Strøm et al., 2002a,b). Similarly, a derived peptide from the tryptophan-rich antimicrobial tridecapeptide indolicidin is highly haemolytic (Strøm et al., 2002b). Furthermore, Subbalakshmi et al. (2000) showed that the haemolytic activity of indolicidin is almost entirely abolished when four Trp are replaced with Leu.

### Antimicrobial Activity Assays

The antimicrobial activity of AMPs is the most decisive index for evaluating the clinical application of antimicrobial drugs. Many antimicrobial agents inhibit bacterial growth rather than kill the bacteria quickly, and long-term infections are pathogens that are not completely killed, which results in bacterial severe drug resistance. Thus, as shown in Table 2, the antimicrobial efficacies of the WR and LR series against common bacterial strains were articulated with the minimum inhibitory/bactericidal concentrations (MICs/MBCs). The geometric mean (GM) values of the MICs/MBCs of the two series across all of the tested bacterial strains are represented in Table 3, which depicts the average bacteriostatic/bactericidal level of a peptide. The results showed that the MBC values of almost all WR and LR series peptides were 2–4 times higher than the MIC values.

**TABLE 2:** MICs^*a*^ (MBCs^*b*^) (μM) of the WR and LR peptides against bacteria strains.

**Peptide**	**Gram-negative bacteria**										**Gram-positive bacteria**							
	***E. coli* 25922**	***E. coli* UB1005**	***S. typhi -murium* 14028**	***S. pullorum* 7913**	***P. aeruginosa* 27853**	***E. coli* K88**	***E. coli* K99**	***E. coli* O78**	***S.typhi -murium* 7731**	**GM _*MIC/MBC*_**	***S. aureus* 25923**	***S. aureus* 29213**	***S. epidermidis* 12228**	***MRSA*^*c*^ 43300**	***B. subtilis* 63501**	***E. faecalis* 29212**	***Listeria monocy -togenes* cgmcc 1.1075**	**GM _*MIC/MBC*_**
W1	4 (8)	4 (8)	4 (8)	4 (8)	8 (16)	8 (16)	4 (16)	4 (8)	4 (8)	4.67/10.08	2 (4)	2 (4)	2 (4)	2 (4)	1 (2)	4 (8)	8 (16)	2.44/4.88
W2	4 (8)	4 (8)	8 (16)	4 (8)	16 (32)	4 (8)	4 (16)	8 (16)	4 (8)	5.44/11.76	2 (4)	2 (4)	2 (4)	2 (4)	1 (2)	8 (16)	16 (32)	2.97/5.94
W3	4 (8)	4 (8)	4 (8)	2 (4)	4 (8)	4 (8)	16 (32)	4 (8)	8 (16)	4.67/9.33	4 (8)	4 (8)	2 (4)	2 (4)	1 (2)	4 (8)	4 (8)	2.69/5.38
W4	4 (8)	8 (16)	8 (16)	4 (8)	8 (16)	4 (8)	4 (8)	8 (16)	8 (16)	5.88/11.76	2 (4)	2 (4)	2 (4)	2 (4)	1 (2)	4 (8)	16 (32)	2.69/5.38
W5	2 (4)	2 (4)	4 (8)	2 (4)	4 (8)	2 (4)	2 (4)	4 (8)	4 (8)	2.72/5.44	2 (4)	2 (4)	2 (4)	2 (4)	1 (2)	4 (8)	2 (4)	2.00/4.00
W6	4 (8)	4 (8)	8 (16)	8 (16)	8 (16)	4 (8)	2 (4)	4 (8)	8 (16)	5.04/10.08	2 (4)	2 (4)	4 (8)	2 (4)	1 (2)	4 (8)	2 (4)	2.21/4.42
W7	4 (8)	4 (8)	8 (16)	4 (8)	4 (32)	4 (8)	8 (16)	8 (16)	16 (32)	5.88/13.72	2 (4)	4 (8)	2 (4)	2 (4)	1 (2)	8 (16)	2 (4)	2.44/4.88
W8	4 (8)	4 (8)	4 (8)	2 (4)	4 (8)	4 (8)	2 (4)	4 (8)	4 (8)	3.43/6.86	2 (4)	2 (4)	2 (4)	2 (4)	1 (2)	4 (8)	2 (4)	2.00/4.00
W9	4 (8)	4 (8)	8 (16)	4 (8)	8 (16)	4 (8)	4 (8)	4 (8)	4 (8)	4.67/9.33	2 (4)	2 (4)	2 (4)	2 (4)	2 (4)	4 (8)	4 (8)	2.44/4.88
W10	8 (16)	8 (16)	8 (16)	16 (32)	4 (8)	4 (8)	2 (4)	4 (8)	4 (8)	5.44/10.89	2 (4)	2 (4)	2 (4)	2 (4)	2 (4)	4 (8)	2 (4)	2.21/4.42
W11	4 (8)	4 (8)	8 (16)	4 (8)	8 (16)	4 (8)	8 (16)	8 (16)	8 (16)	5.88/11.76	2 (4)	2 (4)	2 (4)	2 (4)	1 (2)	4 (8)	4 (8)	2.21/4.42
W12	4 (8)	4 (8)	4 (8)	4 (8)	4 (8)	8 (16)	2 (4)	4 (8)	8 (16)	4.32/8.64	2 (4)	2 (4)	2 (4)	2 (4)	1 (2)	4 (8)	2 (4)	2.00/4.00
L1	8 (16)	16 (32)	16 (32)	8 (16)	> 64	8 (16)	8 (16)	8 (16)	16(32)	13.72/25.40	32 (64)	16 (32)	16 (32)	16 (32)	2 (4)	64 (128)	> 64	21.53/39.01
L2	8 (16)	16 (32)	64 (128)	16 (32)	> 64	16 (32)	8 (16)	8 (16)	64(128)	21.77/40.32	> 64	64 (128)	> 64	32 (64)	2 (4)	> 64	>64	52.50/70.66
L3	8 (16)	16 (32)	64 (128)	8 (16)	64 (128)	8 (16)	16 (32)	16 (32)	32 (64)	18.66/37.33	> 64	>64	> 64	>64	8 (16)	> 64	16 (32)	64.00/78.02
L4	> 64	>64	> 64	>64	> 64	>64	> 64	>64	> 64	128.00/128.00	> 64	>64	> 64	>64	8 (16)	> 64	>64	86.14/95.10
L5	2 (4)	4 (8)	4 (8)	2 (4)	8 (16)	2 (4)	4 (8)	2(4)	4 (8)	3.17/6.35	8 (16)	8 (16)	8 (16)	8 (16)	1 (2)	16 (32)	4 (8)	5.94/11.89
L6	> 64	>64	> 64	>64	> 64	64 (128)	64 (128)	64 (128)	> 64	101.59/128.00	> 64	>64	> 64	>64	32 (64)	> 64	>64	105.00/115.93
L7	> 64	>64	> 64	>64	> 64	32 (64)	64 (128)	64 (128)	> 64	94.06/118.51	> 64	>64	> 64	>64	8 (16)	> 64	16 (32)	64.00/78.02
L8	16 (32)	32 (64)	32 (64)	8 (16)	64 (128)	4 (8)	16 (32)	8 (16)	32 (64)	17.28/32.00	64 (128)	32 (64)	32 (64)	32 (64)	4 (8)	> 64	4 (8)	23.78/43.07
L9	16 (32)	64 (128)	64 (128)	32 (64)	> 64	16 (32)	16 (32)	32 (64)	64(128)	37.33/69.12	> 64	>64	> 64	>64	8 (16)	> 64	16 (32)	64.00/78.02
L10	32 (64)	> 64	>64	> 64	32 (64)	32 (64)	4 (8)	16 (32)	64(128)	40.32/64.00	> 64	>64	> 64	>64	16 (32)	> 64	16 (32)	70.66/86.14
L11	> 64	>64	> 64	>64	> 64	32 (64)	> 64	64 (128)	> 64	101.59/118.51	> 64	>64	> 64	>64	8 (16)	> 64	8 (16)	57.97/70.66
L12	32 (64)	32 (64)	> 64	32 (64)	> 64	16 (32)	> 64	64 (128)	> 64	59.26/87.09	> 64	>64	> 64	>64	16 (32)	> 64	16 (32)	70.66/86.14
Melittin	4 (8)	4 (8)	4 (8)	4 (8)	16 (32)	2 (4)	4 (8)	4 (8)	4 (8)	4.32/8.64	2 (2)	4 (8)	2 (4)	2 (4)	2 (4)	2 (2)	0.5 (0.5)	1.81/3.28
Polymyxin B	4 (8)	2 (4)	4 (8)	4 (8)	8 (16)	2 (4)	2 (4)	2 (4)	2 (4)	2.94/5.88	2 (4)	4 (8)	2 (4)	2 (4)	1 (2)	32 (> 64)	32 (> 64)	4.42/10.77

**^*a*^MIC (μM) was determined as the lowest concentration of peptide that inhibited 95% of the bacterial growth. Data are representative of three independent experiments. ^*b*^MBCs (μM) were determined as the lowest peptide concentration that killed greater than 99.9% of the bacterial cells. The data were derived from representative values of three independent experimental trials. ^*c*^Methicillin-resistant S. aureus.**

**TABLE 3:** MHC, GM, and TI values of the WR and LR peptides.

**Peptide**	**MHC^*a*^**	**GM^*b*^**			**TI^*c*^**		
		**Gram-negative bacteria**	**Gram-positive bacteria**	**All**	**Gram-negative bacteria**	**Gram-positive bacteria**	**All**
W1	64	4.67	2.44	3.51	13.70	26.23	18.23
W2	> 128	5.44	2.97	4.18	47.06	86.20	61.24
W3	> 128	4.67	2.69	3.67	54.82	95.17	69.75
W4	128	5.88	2.69	4.18	21.77	47.58	30.62
W5	> 128	2.72	2.00	2.38	94.12	128.00	107.56
W6	> 128	5.04	2.21	3.51	50.79	115.84	72.93
W7	> 128	5.88	2.44	4.00	43.54	104.92	64.00
W8	128	3.43	2.00	2.71	37.32	64.00	47.23
W9	> 128	4.67	2.44	3.51	54.82	104.92	72.93
W10	> 128	5.44	2.21	3.67	47.06	115.84	69.75
W11	> 128	5.88	2.21	3.83	43.54	115.84	66.84
W12	128	4.32	2.00	3.08	29.63	64.00	41.56
L1	> 128	13.72	21.53	16.71	18.66	2.97	3.83
L2	> 128	21.77	52.50	33.42	11.76	4.88	7.66
L3	> 128	18.66	64.00	32.00	13.72	4.00	8.00
L4	> 128	128.00	86.14	107.63	2.00	2.97	2.38
L5	> 128	3.17	5.94	4.18	80.76	43.10	61.24
L6	> 128	101.59	105.00	103.07	2.52	2.44	2.48
L7	> 128	94.06	64.00	79.48	2.72	4.00	3.22
L8	> 128	17.28	23.78	19.87	14.81	10.77	12.88
L9	> 128	37.33	64.00	47.26	6.86	4.00	5.42
L10	> 128	40.32	70.66	51.54	6.35	3.62	4.97
L11	> 128	101.59	57.97	79.48	2.52	4.42	3.22
L12	> 128	59.26	70.66	64.00	4.32	3.62	4.00
Melittin	1	4.32	1.81	2.95	0.23	0.55	0.34

**^*a*^MHC is the minimum hemolytic concentration that caused 10% hemolysis of hRBCs. Data are representative of three independent experiments. When no detectable hemolytic activity was observed at 128 μM, a value of 256 μM was used to calculate the TI. ^*b*^The GM of the peptide MICs against bacteria was calculated. When no detectable antimicrobial activity was observed at 64 μM, a value of 128 μM was used to calculate the TI. ^*c*^TI is calculated as MHC/GM. Larger values indicate greater therapeutic potential.**

As shown in Table 3, GMs of the LR peptides ranged from 4.18 to 107.63 μM. Our preliminary results suggest that the sequences (order of amino acids) have a considerable impact on the activity. Furthermore, for the LR peptides, only L5 and L8, which can form a stable β-sheet structure in SDS micelles or POPC/POPG SUVs, have strong antibacterial activity. Especially, peptide L5 which can form a nicely amphipathic β-sheet is highly active, usually at least four times more active than any other peptides. Unlike LR peptides, the GMs of the WR peptides ranged from 2.38 to 4.18 μM. It is found that for the WR peptides, the sequence (order of amino acids) has a small effect on the activity—any order gives similar MIC values. It is worth noting that most WR peptides can form a stable secondary structure in either PBS or membrane-mimetic environment. It may be the leading cause of the similar antibacterial activity of WR peptides. Therefore, it has been well documented that a stabilized secondary structure in a membrane-mimetic environment is critically vital for membrane-lytic bioactivity of peptides (Takahashi et al., 2010; Wiradharma et al., 2011; Wang et al., 2018).

Also, it came to our attention that peptides containing Trp and Arg had better activity than those containing Leu and Arg. It has been well demonstrated that Arg- and Trp-rich AMPs have relatively strong activity even at extremely short peptide lengths (Zhu et al., 2014). This phenomenon is perhaps not surprising, because tryptophan displays a strong preference for the lipid membrane interface region when incorporated into biological membranes (Hu et al., 1993; Yau et al., 1998; Esbjörner et al., 2007). Thanks to the presence of electronegative π-electron clouds above or below the aromatic ring, Trp can interact with side chains of cationic residues as well as amino choline head group of the lipid bilayer, which is described as cation-π interaction (Dougherty, 1996). Hence, Arg and Trp can participate in cation–π interactions, which are crucial for peptide self-association in membranes and promote deeper insertion into membranes by sheltering cationic side chains (Gallivan and Dougherty, 1999). Furthermore, this effect is also attributed to the bulkiness of the indole ring of Trp, disrupting the hydrophobic interactions among the lipid acyl chains (Yau et al., 1998).

As we mentioned above, it is still inappropriate to study the relationship between amphipathicity and activity by keeping key parameters constant and only changing the order of the amino acids in the sequence. Because changing the amino acid sequence may also lead to changes in the secondary structure of the peptides, which just happens to be confirmed by our peptide CD spectrum. Therefore, after changing the order of the amino acids, it is significant to screen out peptides with a stable secondary structure to investigate the relationship between amphiphilicity and antimicrobial activity of AMPs. For LR peptides, only L5 and L8 are capable of forming a β-sheet amphipathic structure. As for WR peptides, most of them are able to form amphipathic β-sheet structure mainly. Therefore, the β-sheet hydrophobic moment was referred to study the relationship between the activity of the β-sheet peptides and the amphiphilicity. The results show that whether it is WR or LR peptides, the higher the β-sheet hydrophobic moment, the better the antibacterial activity of the AMPs. Especially, L5, L8, W5, and W8, which have relatively high hydrophobic moments compared with other peptides in their respective series, have nicely amphipathic β-sheet and antimicrobial activity.

Finally, we found that W5 and L5, which had the same order of amino acids, formed a nicely amphipathic β-sheet and had the best antibacterial activity simultaneously. The MICs of W5 (GM_*all*_ = 2.38 μM) and L5 (GM_*all*_ = 4.18 μM) were better or close to that of melittin (GM_*all*_ = 2.95 μM). Moreover, the therapeutic index (TI = MHC/GM_*MIC*_) reflected the biocompatibility degree of a peptide and was used to evaluate the clinical application potential of the AMPs. In the WR and LR series (Table 3), W5 (TI_*all*_ = 107.56) and L5 (TI_*all*_ = 61.24) also had the highest TI values within their respective series, which showed excellent therapeutic potential compared with melittin (TI_*all*_ = 0.34) and other peptides of the same series. These results show that nicely amphipathic β-sheet peptides have great cell selectivity, suggesting that possessing significant antimicrobial activities and low hemolytic simultaneously.

**FIGURE 5:**
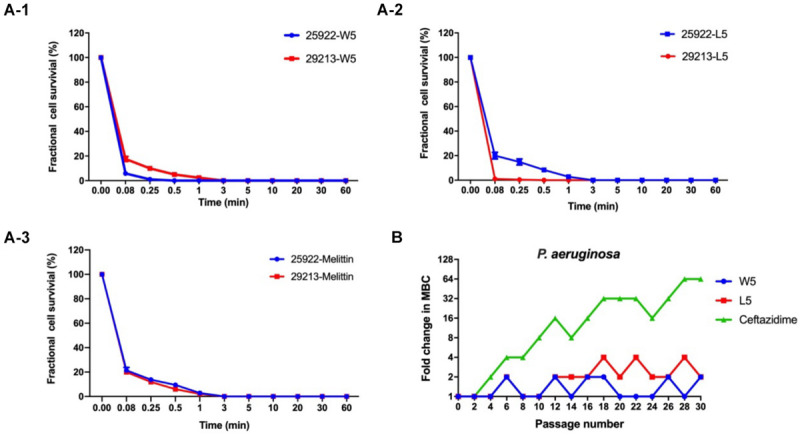
**(A)** Time-kill kinetic curves of W5 **(A-1)**, L5 **(A-2)**, and melittin **(A-3)** at 1× MBC against *E. coli* ATCC 25922 and *S. aureus* 29213. **(B)** Resistance proceedings in the presence of a sub-MBC concentration of the peptides against *P. aeruginosa* ATCC 27853. The graph was from three independent experiments, and each independent experiment contained three technical replicates.

Hence, W5 and L5, as the best candidates among the WR and LR series, were further investigated for their efficiency to kill bacteria. Melittin was employed as a positive control to determine its killing kinetics. W5 and L5, at 1 × MIC concentration, consecutively increased the exposure time of *Escherichia coli* 25922 (*E. coli* 25922) and *Staphylococcus aureus* 29213 (*S. aureus* 29213). The results from Figure 5A signified that W5 and L5 had fast sterilization efficiency. W5 and L5 obliterated the growth of 99.99% of *E. coli* 25922 within 0.25 and 3 min. They cleared off the growth of 99.99% of *S. aureus* 29213 within 3 and 0.08 min, respectively, compared with melittin, which obliterated greater than 99.99% of the bacterial cells within 3 min.

Long-term exposure of microorganisms to sublethal concentrations of drugs could facilitate the emergence of resistance (DžIdic´ et al., 2008). Hence, a drug resistance assay was performed to evaluate whether bacteria quickly evolved resistance to W5 and L5 (Figure 5B). After 30 sequential passages of *P. aeruginosa* 27853 in sub-MBC concentrations of W5 and L5, the strain maintained great sensitivity to W5 and L5, which suggested that resistance to W5 and L5 was minimal. In comparison, ceftazidime stimulation immediately showed resistance at the 10th passage and increased the MBC value 64-fold after 30 passages, suggesting that resistance to antibiotics was generated quickly with subtherapeutic treatment.

### Salt Sensitivity Assays

To adequately evaluate the clinical application potentials of the WR and LR series, their antimicrobial activities in the presence of various salts were determined. As shown in Tables 4, 5, compared to their MICs, the antimicrobial activities of most peptides against *E. coli* 25922 and *S. aureus* 29213 were partially decreased in the presence of the cations of Na^+^, K^+^, Ca^2+^, and Mg^2+^. In particular, Na^+^ and Ca^2+^ severely impaired the antimicrobial activity of the majority of peptides, with MICs increasing by 2–8-fold. A possible reason for this result was that salts, such as Na^+^ and Ca^2+^, in the surrounding medium could impede electrostatic interactions and reduce binding of AMPs to the bacterial membrane by the charge screening effect (Fedders et al., 2008). Divalent or multivalent cations could also compromise the activity of AMPs by competing for membranes and combining peptides and cations (Huang et al., 2011). Furthermore, we found that other cations (NH_4_^+^, Zn^2+^ and Fe^3+^) had a slight effect or even a motivating impact on the antimicrobial activities. It is widely known that the effects of cations might be peptide-dependent or concentration-dependent (Aquila et al., 2013; Ma et al., 2016); for example, a low concentration of salt can improve the activity of AMPs, but a higher concentration of cations increases membrane rigidity, which slowly hinders pore formation. Consistent with the above studies, our results showed that higher concentrations of Na^+^ (150 mM), K^+^ (4.5 mM), Ca^2+^ (2 mM), and Mg^2+^ (1 mM) compromised the activity of the AMPs. Still, low concentrations of NH_4_^+^ (6 μM), Zn^2+^ (8 μM), and Fe^3+^ (4 μM) increased or had little effect on their activity.

**TABLE 4:** MIC values of WR and LR peptides against *E. coil* ATCC 25922 in the presence of physiological salts.

**Peptide**	**Control^*a*^**	**NaCl^*a*^**	**KCl^*a*^**	**CaCl_2_^*a*^**	**MgCl_2_^*a*^**	**ZnCl_2_^*a*^**	**FeCl_3_^*a*^**	**NH_4_Cl^*a*^**
W1	4	8	4	2	2	2	2	4
W2	4	16	4	8	4	4	8	4
W3	4	16	8	8	8	8	8	4
W4	4	16	16	8	16	4	4	2
W5	2	4	4	4	2	4	4	4
W6	4	16	8	16	8	4	4	2
W7	4	8	8	8	4	4	8	4
W8	4	32	16	8	16	4	2	4
W9	4	16	8	16	8	8	4	4
W10	8	32	16	16	16	4	4	8
W11	4	8	8	16	8	2	4	2
W12	4	32	4	16	4	2	4	4
L1	8	64	16	32	8	8	4	4
L2	8	32	16	32	16	8	8	8
L3	8	32	8	32	32	16	16	16
L4	> 64	>64	> 64	>64	> 64	>64	> 64	>64
L5	2	4	0.5	2	0.5	0.5	0.5	0.5
L6	> 64	>64	> 64	>64	> 64	>64	> 64	>64
L7	> 64	>64	> 64	>64	> 64	>64	> 64	>64
L8	16	64	32	> 64	32	8	16	8
L9	16	> 64	16	> 64	32	16	16	32
L10	32	32	32	32	32	32	32	32
L11	> 64	>64	> 64	>64	> 64	>64	> 64	>64
L12	32	> 64	32	> 64	>64	32	32	32
Melittin	2	16	16	8	4	2	2	2
Polymyxin B	4	8	8	8	4	4	4	4

**^*a*^The final concentrations of NaCl, KCl, NH_4_Cl, MgCl_2,_ CaCl_2_, ZnCl_2_, and FeCl_3_ were 150 mM, 4.5 mM, 6 μM, 1 mM, 2 mM, 8 μM, and 4 μM, respectively, and the control MIC values were determined in the absence of these physiological salts. The data were derived from representative value of three independent experimental trials.**

**TABLE 5:** MIC values of the WR and LR peptides against *S. aureus* 29213 in the presence of physiological salts.

**Peptide**	**Control^*a*^**	**NaCl^*a*^**	**KCl^*a*^**	**CaCl_2_^*a*^**	**MgCl_2_^*a*^**	**ZnCl_2_^*a*^**	**FeCl_3_^*a*^**	**NH_4_Cl^*a*^**
W1	2	4	2	2	2	2	2	2
W2	2	4	4	4	4	4	4	2
W3	4	8	2	4	4	4	8	8
W4	2	1	1	2	1	1	2	1
W5	2	2	4	2	4	2	1	2
W6	2	4	2	4	4	2	2	2
W7	4	4	4	4	4	4	8	4
W8	2	2	1	4	4	4	2	4
W9	2	2	2	4	2	2	2	2
W10	2	2	2	2	2	1	1	1
W11	2	2	1	2	2	2	2	1
W12	2	4	2	2	2	2	2	2
L1	32	> 64	64	> 64	64	64	> 64	64
L2	> 64	>64	> 64	>64	> 64	>64	> 64	>64
L3	> 64	>64	> 64	>64	> 64	>64	> 64	>64
L4	> 64	>64	> 64	>64	> 64	>64	> 64	>64
L5	8	64	8	16	8	8	8	8
L6	> 64	>64	> 64	>64	> 64	>64	> 64	>64
L7	> 64	>64	> 64	>64	> 64	>64	> 64	>64
L8	32	> 128	64	> 64	64	32	32	32
L9	> 64	>64	> 64	>64	> 64	>64	> 64	>64
L10	> 64	>64	> 64	>64	> 64	>64	> 64	>64
L11	> 64	>64	> 64	>64	> 64	>64	> 64	>64
L12	> 64	>64	> 64	>64	> 64	>64	> 64	>64
Melittin	4	8	4	8	4	4	4	4
polymyxin B	16	32	16	64	16	16	16	16

**^*a*^The final concentrations of NaCl, KCl, NH_4_Cl, MgCl_2,_ CaCl_2_, ZnCl_2_, and FeCl_3_ were 150 mM, 4.5 mM, 6 μM, 1 mM, 2 mM, 8 μM, and 4 μM, respectively, and the control MIC values were determined in the absence of these physiological salts. The data were derived from representative value of three independent experimental trials.**

Besides, the MICs of the WR series only increased 2–4-fold against *E. coli* 25922 and had little effect or even a slight stimulatory effect against *S. aureus* 29213 in various physiological salt concentrations. In contrast, the MICs of the LR series increased to 2–8-fold against *E. coli* 25922 and were almost inactive against *S. aureus* 29213 in the presence of physiological Na^+^ and Ca^2+^ concentrations. Some studies have shown that Trp-rich AMPs have strong salt resistance due to their ability to position themselves deeply into microbial cell membranes and disrupt the membranes more efficiently (Yu et al., 2013). It is the reason that these salts only have a small effect on the antimicrobial activity of the WR series.

Interestingly, after adding salts, the antimicrobial activities of the WR series were weakly reduced against the gram-negative strain (*E. coli* 25922). Still, the bacteriostasis had almost no effect on the gram-positive strain (*S. aureus* 29213). This phenomenon could be explained by the fact that added cations compete with cationic AMPs to bind to lipopolysaccharide (LPS) (Michael and Yount, 2003). Although lipoteichoic acids carry negative charges, cations may not combine with them. It is satisfactory that various salts have little effect on the antimicrobial activity of W5 and L5, which still maintain high antimicrobial activity in solutions of Na^+^ and Ca^2+^.

### Preliminary Mechanistic Studies

Under the above results, W5 and L5 were deemed to have the optimum antimicrobial performance and cell selectivity against all tested strains. Therefore, W5 and L5 were chosen for study on the bactericidal mechanism.

**FIGURE 6:**
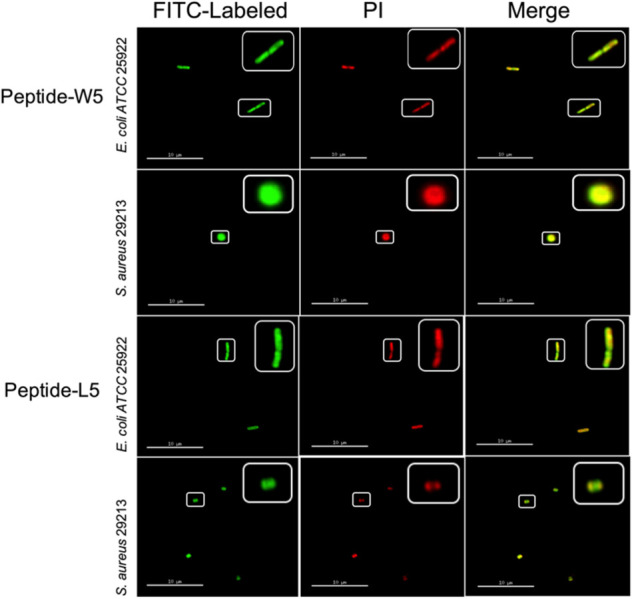
Super-resolution microscopy image analysis of *S. aureus* 29213 and *E. coli* ATCC 25922 treated with PI, FITC-labeled W5 and FITC-labeled L5. The green signal is from the FITC peptides, and the red signal is from PI. The FITC peptides mainly cover the surface of the membrane, and PI is a nucleic acid dye that can penetrate broken cells and release red fluorescence.

First, to directly observe the location of peptides on the bacteria, super-resolution microscopy was used with the addition of PI, which could release red fluorescence, and fluorescein isothiocyanate (FITC)-labeled W5 and L5, which emits green fluorescence. PI is a membrane-impermeable dye that can penetrate damaged cell membranes and stain nuclei. Fluorescence intensity analysis showed that both FITC-W5 and FITC-L5 surrounded the surface of *E. coli* and *S. aureus* (Figure 6). Moreover, the fluorescence released by the red nucleic acid dye PI was observed, which concluded that W5 and L5 were not only located in the bacterial membrane but also killed *E. coli* and *S. aureus* successfully.

According to the peptide localization that was detected and the death of the bacteria, we speculated that L5 and W5 might first combine with LPS by an electrostatic attraction and be drawn to the membrane surface. Then the hydrophobic surface of peptides could be inserted into the phospholipid bilayer of the outer membrane and inner membrane, disturbing the surface of the bacterial membrane and changing the potential of the bacterial membrane. Subsequently, the pores/ion channels created on the cytoplasmic membrane would destroy the integrity of the bacterial membrane and lead to cell death (Epand et al., 2010; Fjell et al., 2012; Sharma et al., 2016; Dou et al., 2017).

**FIGURE 7:**
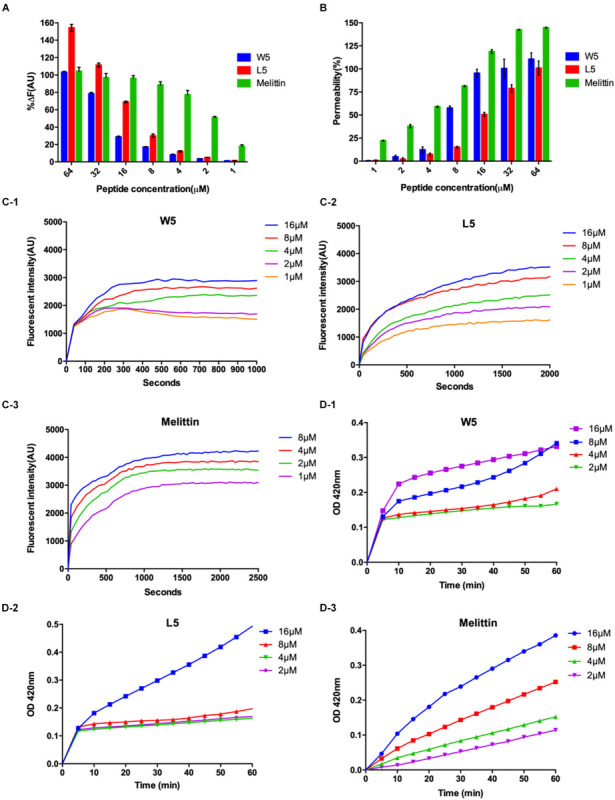
**(A)** Peptide binding affinity to LPS from *E. coli* O111:B4. The fluorescence intensity was monitored at an excitation wavelength of 580 nm and an emission wavelength of 620 nm. **(B)** The outer membrane permeability induced by W5, L5, and melittin. The uptake of NPN by *E. coli* in the presence of different concentrations of W5, L5 and melittin was determined using the fluorescent dye (NPN) assay. The NPN uptake was monitored at an excitation wavelength of 350 nm and an emission wavelength of 420 nm. **(C)** The cytoplasmic membrane potential variation of *E. coli* treated with different concentrations of W5 **(C-1)**, L5 **(C-2)**, or melittin **(C-3)**, as assessed by the release of the membrane potential-sensitive dye DiSC_3_-5. The fluorescence intensity was monitored at an excitation wavelength of 622 nm and an emission wavelength of 670 nm as a function of time. **(D)** Inner membrane permeability of the peptides. The hydrolysis of ONPG due to the release of cytoplasmic β-galactosidase by *E. coli* treated with different concentrations of W5 **(D-1)**, L5 **(D-2)**, or melittin **(D-3)** was measured spectroscopically at an absorbance of 420 nm as a function of time. The graphs were derived from the average of three independent trials.

To confirm this assumption, we measured the combining capacity of W5 and L5 to LPS by employing the fluorescence displacement method of BODIPY-TR cadaverine. LPS, a vital ingredient of the external leaflet of the gram-negative bacterial outer membrane, protects bacteria from various host defense invasions (Avitabile et al., 2013). Furthermore, LPS combines with peptides via electrostatic interactions to regulate membrane insertion. As shown in Figure 7A, the binding capacity of W5 and L5 to LPS showed a dose-dependent increase that was similar to that of melittin. The binding ability of L5 to LPS was more potent than that of W5 at all concentrations due to the steric hindrance of the more massive indole ring of Trp. The LPS binding efficiency of W5 and L5 showed significant boosts at 32 and 16 μM, respectively, reaching nearly 80%. In addition, the LPS binding ability of L5 was stronger than that of melittin when the concentration was greater than 32 μM.

Next, the outer membrane permeability of W5 was detected via the fluorescence of 1-N-phenylnapthylamine (NPN), a hydrophobic fluorescence probe. NPN can increase the fluorescence intensity if it enters a cell whose outer membrane is disturbed. The bacterial outer membrane permeabilization of W5 and L5 was satisfactory and still presented in a dose-dependent manner. In contrast to the binding ability to LPS, the outer membrane permeabilization of W5 was higher than that of L5 at all concentrations. As shown in Figure 7B, W5 reached approximately 100% permeabilization at 16 μM, which was similar to the permeabilization of melittin, whereas the outer membrane permeabilization of L5 only reached approximately 50% at the same concentration. Studies have shown that LPS may diminish the permeability of bacterial membranes. Accordingly, we speculated that the strong binding ability of L5 to LPS led to lower permeability of the outer membrane compared with W5. These results indicated that W5 and L5 could combine with LPS and permeabilize the outer membrane.

Subsequently, DiSC_3_-5, a membrane potential-dependent probe, was used to determine whether W5 and L5 could disturb the cytoplasmic membrane potential. Usually, DiSC_3_-5 gathers inside a cell with no fluorescence. Once the cytoplasmic membrane is disrupted or damaged due to changing ion flux, the membrane potential dissipates. Consequently, DiSC_3_-5 is released into the medium, increasing in the fluorescence. Figure 7C shows the membrane potential variations of *E. coli* after the addition of diverse concentrations of W5, L5 and melittin. The fluorescence intensities (AU values) of W5, L5 and melittin displayed dose- and time-dependence within 1,000, 2,000 and 2,500 s, respectively. With an increase in peptide concentration, the cytoplasmic membrane depolarization level of *E. coli* induced by L5 and W5, increased but was still less than that caused by melittin. Furthermore, compared with L5 and melittin, which achieved the highest fluorescence intensity between 1,000 and 1,500 s, W5 produced quick cytoplasmic membrane depolarization within 300 s at all concentrations. The above results indicated that W5 and L5 displayed greater depolarization capacity of the cytoplasmic membrane potential. The cytoplasmic membrane permeabilities of W5, L5 and melittin were further estimated by measuring the release of cytoplasmic β-galactosidase. Once o-nitrophenyl-β-D-galactopyranoside (ONPG) enters the damaged cytoplasmic membrane, β-galactosidase decomposes ONPG into o-nitrophenol and generates an absorbance at 420 nm. As shown in Figure 7D, a general dose-dependent relationship was observed. Additionally, W5 and L5 led to a fast release of cytoplasmic β-galactosidase at 2 μM, and the membrane permeability was greater than that of melittin at low concentrations. Interestingly, the permeability of L5 did not significantly change below 8 μM, whereas it increased rapidly to 16 μM. Taken together, the above results indicated that W5 and L5 penetrated the *E. coli* outer membrane in a concentration-dependent manner. Then, we confirmed that W5 and L5 exhibited the capability allow penetration of the inner membrane to ONPG at 2 μM, and the potential of the cytoplasmic membrane was simultaneously dispersed due to membrane interruption, thereby leading to cytoplasmic content leakage.

**FIGURE 8:**
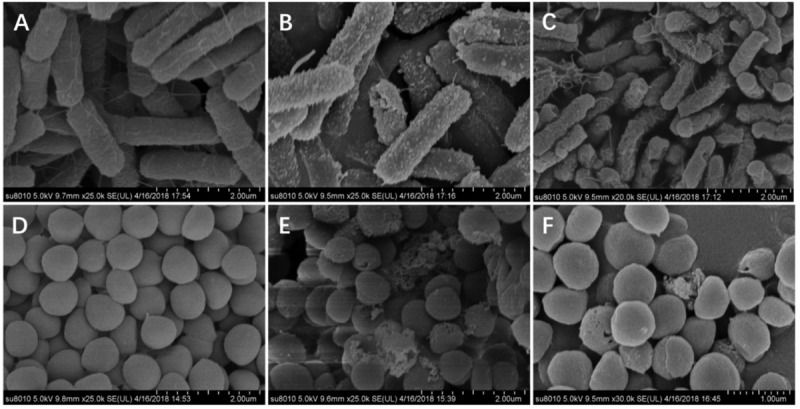
SEM micrographs of *E. coli* ATCC 25922 and *S. aureus* 29213. **(A)** *E. coli*-control, **(B)** *E. coli* treated with W5 (blebbing), **(C)** *E. coli* treated with L5 (hole and wrinkle formation), **(D)** *S. aureus* control, **(E)** *S. aureus* treated with W5 (content leakage), and **(F)** *S. aureus* treated with L5 (hole formation). Scale bar = 2.00 μm **(A–E)**, 1.00 μm **(F)**.

Next, SEM (Figure 8) was used to visually observe the cell morphology and membrane integrity after peptide treatment. Compared to the control *E. coli* with intact and smooth surface (Figures 8A,D), the *E. coli* membrane surface-displayed numerous blebs after treatment with W5 at 1 × MBC for 1 h (Figure 8B). Treatment with L5 (Figure 8C) induced distinctive *E. coli* membrane rupture and pore formation. Furthermore, treatment of *S. aureus* with W5 and L5 was also conducted. Compared with normal *S. aureus* (Figure 8D), the membrane surface of *S. aureus* treated with W5 (Figure 8E) exhibited marked pore formation and isolated cell fragments, and the bacterial surface interacted with L5 (Figure 8F) and presented pores that generated a great deal of intracellular content leakage from the bacteria.

**FIGURE 9:**
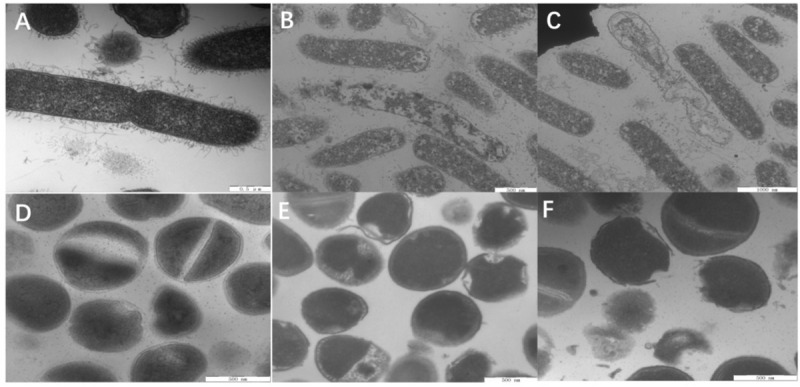
TEM micrographs of *E. coli* ATCC 25922 and *S. aureus* 29213. **(A)** *E. coli* control, **(B)** *E. coli* treated with W5 (content leakage), **(C)** *E. coli* treated with L5 (obviously clear areas), **(D)** *S. aureus* control, **(E)** *S. aureus* treated with W5 (sparse cytoplasmic distribution), and **(F)** *S. aureus* treated with L5 (cytoplasmic membrane and outer membrane separation and hole formation). Scale bar = 500 nm **(A,B,D–F)**, 1,000 nm **(C)**.

Last, the membrane morphologies and intracellular ultrastructural variations in *E. coli* and *S. aureus* after W5 and L5 treatment at 1 × MBC for 1 h were observed by TEM (Figure 9). Compared with untreated *E. coli* (Figure 9A), *E. coli* treated with W5 showed sparse cytoplasmic distribution and content leakage (Figure 9B). *E. coli* treated with L5 displayed creping and clear areas (Figure 9C). In *S. aureus*, treatment with W5 (Figure 9E) and L5 (Figure 9F) at 1× MBC for 1 h caused clear outer and inner membrane segregation and pore formation, which led to intracellular content leakage, thus resulting in a thin cytoplasmic dispersion and distinct clear areas compared with that of the control.

Overall, L5 and W5 first interacted with LPS via electrostatic adsorption. When the peptides aggregated and reached the threshold, the hydrophobic cores of W5 and L5 inserted into the outer membrane. Subsequently, W5 and L5 continued to interact with the cytoplasmic membrane. Due to insertion into the cytoplasmic membrane, pore/ion channel formation, which is accompanied by potential membrane disruption, occurs (Epand et al., 2010; Fjell et al., 2012). Fluorescence microscopy and field emission SEM and TEM analysis visually illustrated that W5 and L5 could penetrate the cell wall and cell membrane, indicating that W5 and L5 relied on a membrane-disruptive mechanism.

## Conclusion

In this study, two series of peptides containing 4 Arg and 5 Trp or 5 Leu were designed only by changing the order of amino acids in the sequence. Under CD spectra results, we found that different amino acids order had a significant impact on the secondary structure of LR peptides but little effect on WR peptides. According to the results of biocompatibility, it was found that different amino acids order almost had no impact on the host cytotoxicity of WR and LR peptides, on the contrary, amino acid types had a more significant impact on hemolysis and cytotoxicity. By analyzing the CD spectrum and MIC results, we demonstrated that the formation of a stable secondary structure was crucial for potent antimicrobial activity. For WR peptides, most of them can form stable secondary structures, so almost all of them had great antimicrobial activity. For LR peptides, only L5 and L8, which can form stable β-sheets, possessed high antimicrobial activity. Based on forming nicely amphiphilic β-sheet structure, whether it was WR peptide or LR peptide, high β-sheet amphipathicity was accompanied by high antimicrobial activity. In two series, W5 [(RW)_4_W] and L5 [(RL)_4_L], which shared the same amino acids order (R alternating with W or L) and formed a characteristically amphiphilic β-sheet structure, had the optimal therapeutic index. Moreover, W5 and L5 had rapid sterilization efficiency, high salt tolerance and hardly generated any drug resistance. Furthermore, W5 and L5 exerted bactericidal action by locating the cell membrane surface and penetrating the bacterial outer membrane and cytoplasmic membrane, which caused content leakage and cell death. The findings reported here provide a rationalization for peptide design and optimization, which will be useful for the future development of antimicrobials.

## Experimental Section

### Peptide Synthesis and Sequence Analysis

The designed and fluorescently labeled peptides that were used in the study were synthesized by Sangon Biotech (Shanghai, China), and their actual molecular weights were verified by matrix-assisted laser desorption/ionization time-of-flight mass spectrometry (MALDI-TOF MS; Linear Scientific Inc., United States). Peptide purity (95%) was evaluated by reverse-phase high-performance liquid chromatography (HPLC) on a SHIMADZU Inertsil ODS-SP column (4.6 mm × 250 mm × 5 μm, 214 nm, 60 μL) using a non-linear water/acetonitrile gradient containing 0.1% trifluoroacetic at a flow rate of 1.0 mL/min. The primary structural parameters (positive charge and hydrophobicity) were calculated online using the HeliQuest analysis Web site^1^.

#### Bacterial Strains

Gram-negative bacteria, including *Escherichia coli (E. coli)* ATCC 25922, *E. coli* 078, *E. coli* K88, *E. coli* K99, *Salmonella pullorum (S. pullorum)* C7913, *Pseudomonas aeruginosa (P. aeruginosa)* ATCC 27853, *Salmonella typhimurium (S. typhimurium)* ATCC 14028 and C7731 were kept in our laboratory. *E. coli* UB1005 was kindly provided by the State Key Laboratory of Microbial Technology (Shandong University, China).

Gram-positive bacteria, including *Staphylococcus aureus (S. aureus)* ATCC 29213, *S. aureus* ATCC 25923, *Staphylococcus epidermidis (S. epidermidis)* ATCC 12228, *Bacillus subtilis (B. subtilis)* 63501, *Enterococcus faecalis (E. faecalis)* ATCC 29212 *and methicillin-resistant S. aureus* ATCC 43300 were obtained from the College of Veterinary Medicine, Northeast Agricultural University (Harbin, China). *Listeria monocytogenes* CGMCC 1.1075 was purchased from China General Microbiological Culture Collection Center (CGMCC).

#### Preparation of SUVs

POPC and POPG lipids in chloroform were purchased from Aladdin (Shanghai, China). The appropriate amount of dry phospholipid [3:1 POPC (30.78 mg)/POPG (10.4 mg)] in chloroform was first dried under a nitrogen stream. The lipid was hydrated with 2 mL of 10 mM PBS and vortexed extensively. Then the milky lipid suspension was sonicated in ice-cold water approximately 20 min using an ultrasonic cleaner until the solutions became transparent. Final SUVs concentration used for CD studies was 1.5 mM.

#### CD Measurements

The CD spectra (λ 190-250 nm) of every peptide at an ultimate concentration of 150 μM were recorded in 10 mM PBS, 30 mM SDS micelles in 10 mM PBS and 1.5 mM 3:1 POPC:POPG SUVs in 10 mM PBS on a J-820 spectropolarimeter (Jasco, Tokyo, Japan) using a quartz cell with a 1.0 mm path length. Every sample was measured > 3 times and the average calculated. The captured CD spectra were then converted to the mean residue ellipticity using the following equation:

(1) $$\theta_{M}=\frac{\theta_{o\,b\,s}}{\text{cln}}$$

where *θ*_*M*_ is the mean residue ellipticity (deg cm^2^ dmol^–1^), *θ*_*obs*_ is the observed ellipticity corrected for the buffer at a given wavelength (mdeg), *c* is the peptide concentration (mM), *l* is the path length (mm), and *n* is the number of peptide bonds.

#### Cytotoxicity Assays

The cytotoxicities of the peptides were determined by a modified standard microtiter dilution method using two cell types, the murine macrophage cell line RAW264.7 and intact human red blood cells (hRBCs). Detection of the first cell type was performed by the 3-(4,5-dimethylthiazol-2-yl)-2,5-diphenyltetrazolium bromide (MTT) dye reduction assay, and detection of the last cell type was determined by a hemolysis test (Wang et al., 2015).

Briefly, for the MTT assay, (1.0-2.0) × 10^5^ cells / well were seeded in 96-well plates and then treated with various concentrations of peptides for 24 h at 37 °C in 5% CO_2_. Then, 50 μL of MTT was added to the cell cultures at a final concentration of 0.5 mg/mL; the mixtures were further incubated for 4 h at 37 °C and centrifuged at 1000 g for 5 min, and the supernatants were discarded. Formazan crystals were dissolved in 150 μL of DMSO, and the OD at 570 nm was measured using a microplate reader (TECAN GENios F129004; TECAN, Austria).

Briefly, 1 mL of intact hRBCs was collected and diluted 10-fold with PBS (pH 7.4). Subsequently, identical volumes of an intact hRBCs solution and peptide solutions at various concentrations were mixed in 96-well plates, and the mixtures were incubated for 1 h at 37°C. The plates were centrifuged at 1,000 g for 10 min, and the supernatants were transferred to new 96-well plates. The hRBCs were incubated with PBS alone and 0.1% Triton X-100 alone, which were deemed the negative and positive controls, respectively. The release of hemoglobin was monitored by determining the absorbance at 576 nm using a microplate reader (TECAN GENios F129004; TECAN, Austria). The peptide concentration that resulted in 10% hemolysis was considered the minimal hemolysis concentration (MHC). The percentage of hemolysis was determined using the following formula:

(2) $$\text{percent}\,\text{hemolysis}=\frac{A-A_{0}}{A_{t}-A_{0}}\times 100$$

where A represents the absorbance of the peptide sample at 576 nm and A_0_ and A_*t*_ express 0 and 100% hemolysis determined in 10 mM PBS and 0.1% Triton X-100, respectively. A minimum of three independent experiments was conducted for the assay, and three technical replicates were used in each experiment.

#### Antimicrobial Activity Assays

The antimicrobial activities of the peptides were measured using a method adapted from the National Committee for Clinical Laboratory Standards (NCCLS) with some modifications (Chou et al., 2016). The specific procedure for these assays has been previously described. In brief, the bacterial cells were grown to a mid-logarithmic phase and diluted in MHB to an ultimate concentration of 0.5–1 × 10^6^ CFU mL^–1^. Subsequently, the peptides were consecutively diluted in a 0.2% BSA solution and then added to a bacterial solution in a 96-well plate at identical volumes. After incubation for 24 h at 37°C, the MICs were determined to be the lowest peptide concentration that inhibited 95% of the bacterial growth by monitoring the absorbance at 492 nm using a microplate reader. Subsequently, 50 μL of each incubation mixture was further transferred to agar plates and incubated overnight. The minimum bactericidal concentrations (MBCs) were determined as the lowest peptide concentration that killed greater than 99.9% of the bacterial cells. Every assay was reproduced more than 3 times.

The kinetics of the sterilization activity of the peptide was further evaluated. *E. coli* ATCC 25922 and *S. aureus* ATCC 29213 (at a final concentration of 0.5–1 × 10^6^ CFU mL^–1^) were treated with peptides at a 1× MIC concentration, and aliquots of the mixture were collected at different times to calculate the cell survival rate. The results of the kinetic were the mean values of three independent experiments, and each independent experiment contained three technical replicates.

Finally, *P. aeruginosa* ATCC 27853 was used as the model of the drug resistance study. Overnight cultures of the *P. aeruginosa* cell serially passaged by 100-fold dilution in 2 mL batch cultures every 12 h in MHB that contained the sub-MBC concentration of the drugs. The MBC values of the drugs against every two passage’s cells were tested. As a control, MBCs were also tested using cells that were serially passaged in fresh MHB without drugs. The assay was conducted independently in triplicate with three biological replicates.

#### Salt Sensitivity Assays

The salt sensitivities of the peptides were determined. *E. coli* ATCC 25922 and *S. aureus* ATCC 29213 were incubated in the presence of various ultimate concentrations of physiological salts (150 mM NaCl, 4.5 mM KCl, 6 μM NH_4_Cl, 8 μM ZnCl_2_, 1 mM MgCl_2_, 2mM CaCl_2_, and 4 μM FeCl_3_) according to our previous protocol (Yang et al., 2019). These tests were performed under the MICs, and three independent assays were performed.

#### Localization of FITC-Labeled Peptides

The action sites of the peptides were further confirmed by using FITC-labeled peptides and propidium iodide (PI) and observed by super-resolution microscopy (Shang et al., 2016). Microbial cells (OD _600_ = 0.2) were incubated with FITC-labeled peptides at 1 × MBC for 15 min at 37 °C. Then, the mixture was centrifuged and washed three times with PBS buffer at 1,000 g. The cells were resuspended and incubated with 10 μg mL^–1^ PI in PBS buffer for 15 min at 4°C, and free PI dye was eliminated by centrifugation. A smear was made, and the images were observed using a Deltavision OMX system with 488 and 535 nm bandpass filters for FITC and PI excitation, respectively. Cells without peptides served as the control.

#### LPS Binding Assay

A BODIPY-TR-cadaverine (BC Sigma, United States) displacement assay was used to determine the abilities of the peptides to bind LPS (Ma et al., 2015); in this assay, a probe bound to cell-free LPS was self-quenched but fluoresced upon release into solution. The LPS from *E. coli O111:B4* (50 μg/mL) was incubated with a BODIPY-TR-cadaverine (5 μg/mL) in Tris buffer (50 mM, pH 7.4) for 4 h. Subsequently, equal volumes of the LPS-probe solution and the peptides (1–64 μM) were incubated in a sterile 96-well black plate for 1 h at 37°C, and the fluorescence was measured on a spectrofluorophotometer (the Infinite 200 pro, Tecan, China) at excitation and emission wavelengths of 580 nm and 620 nm, respectively. Each test was performed independently in triplicate. The values were converted to %ΔF (AU) using the following equation:

(3) %ΔF(AU)=[(F-o⁢b⁢sF)0/(F-100F)0]×100

where F_*obs*_ is the observed fluorescence at a given peptide concentration, F_0_ is the initial fluorescence of BC with LPS in the absence of peptides, and F_100_ is the BC fluorescence with LPS cells upon the addition of 10 μg mL^–1^ polymyxin B, a prototype LPS binder serving as the positive control.

#### Permeabilization of Outer Membrane

Mid-log phase *E. coli* ATCC 25922 cells (OD_600_ = 0.2) were incubated with NPN (10 μM) in 5 mM HEPES buffer (pH 7.4, containing 5 mM glucose) for 30 min. The background fluorescence was recorded for subtraction (excitation λ = 350 nm, emission λ = 420 nm) with an F-4500 fluorescence spectrophotometer (Hitachi, Japan). Subsequently, 100 μL of cell suspension was mixed with equal volumes of the peptide solution at concentrations that ranged from 1 to 64 μM in a sterile 96-well black plate. The fluorescence was recorded over time until no further fluorescence increase was detectable. Each test was performed independently in triplicate, and the results were converted to the percentage of NPN uptake using the equation:

(4) NPNuptake(%)=(F-o⁢b⁢sF)0/(F-100F)0×100%

where F_*obs*_ is the observed fluorescence at a given peptide concentration, F_0_ is the initial fluorescence of NPN with microbial cells in the absence of peptide, and F_100_ is the fluorescence of NPN with microbial cells upon the addition of 10 μg/mL polymyxin B (Sigma) (for bacteria) as a positive control.

#### Cytoplasmic Membrane Depolarization Test

Mid-log phase *E. coli* ATCC 25922 cells (OD_600_ = 0.05) were incubated with 0.4 μM DiSC_3_-5 and 100 mM K ^+^ in 5 mM HEPES buffer (pH 7.4, containing 20 mM glucose) until a steady decrease in fluorescence was achieved. The background fluorescence (excitation λ = 622 nm, emission λ = 670 nm) was recorded with an F-4500 fluorescence spectrophotometer (Hitachi, Japan). Subsequently, 2 mL of the cell suspension was added to a 1-cm quartz cuvette and mixed with various concentrations of peptide. The change in fluorescence in C-1, C-2, and C-3 was recorded from 0 to 1,000, 2,000, and 2,500 s, respectively.

#### Determination of Inner Membrane Permeability

*E. coli* 25922 cells were cultured in MHB containing 2% lactose at 37°C until mid-log phase and then harvested and diluted to an OD_600_ = 0.05 in 5 mM HEPES buffer (pH 7.4, containing 20 mM glucose and 1.5 mM ONPG). Subsequently, equal volumes of cell suspension and peptide solution were added to a sterile 96-well plate, and the mixtures were incubated at 37°C. The OD_420_ measurements were recorded every 5 min from 0 to 60 min, reflecting the influx of ONPG into the cells and thus the permeabilization of the inner membrane.

#### SEM and TEM Characterization

For SEM sample preparation, intermediate log-phase *E. coli* ATCC 25922 cells and *S. aureus* ATCC 29213 cells (OD_600_ = 0.02) were treated with peptides at 1 x MBC for 1 h, harvested and fixed with 2.5% (w/v) glutaraldehyde at 4°C overnight. Subsequently, the sample was continuously treated with a graded ethanol series (50, 70, 90, and 100%) for 10 min, 100% ethanol for 15 min, a mixture (v: v = 1:1) of 100% ethanol and tert-butanol for 15 min, and absolute tert-butanol for 15 min. The sample was dehydrated with liquid CO_2_ in a critical point dryer, coated with gold-palladium, and observed using a Hitachi S-4800 SEM.

SEM was used to prepare a microbial suspension treated with a peptide in the same manner. Then, 10 μL of the extract of the microbial suspension was spread on a glass slide and then air-dried. Image results were obtained using a Bioscope atomic force microscope (Bruker, United States).

TEM samples were initially prepared in the same manner as SEM. After prefixation with 2.5% glutaraldehyde at 4°C overnight, the sample was fixed with 2% osmium tetroxide for 70 min and washed twice with PBS (pH 7.2). Subsequently, the samples were continuously treated with a graded ethanol series (50, 70, 90, and 100%) for 8 min, 100% ethanol for 10 min, a mixture (v: v = 1:1) of 100% ethanol and acetone for 15 min, and anhydrous acetone for 15 min. The samples were then immersed in a mixture of 1:1 anhydrous acetone and epoxy resin for 30 min and the pure epoxy resin overnight. Then, the samples were sliced with an ultramicrotome, stained with uranyl acetate and lead citrate, and observed using a Hitachi H-7650 TEM.

#### Statistical Analysis

Statistical analyses were performed using one-way ANOVA and Student’s *t*-test (two-tailed). Data were analyzed using Social Science (SPSS) version 20.0 (Chicago, IL, United States). Quantitative data are expressed as the mean ± SD, and *P* < 0.01 is considered statistically significant.

## Data Availability Statement

The original contributions presented in the study are included in the article/supplementary material, further inquiries can be directed to the corresponding authors.

## Author Contributions

SH and JW designed and conceived the experiments. SH and ZY conducted the primary experiments assay. SH wrote the main manuscript text. WY and JL conducted the additional experiments. ZL and AS supervised the work and revised the final version of the manuscript. All authors have read and approved the final version of the manuscript.

## Conflict of Interest

The authors declare that the research was conducted in the absence of any commercial or financial relationships that could be construed as a potential conflict of interest.

## Footnotes

1 http://heliquest.ipmc.cnrs.fr/cgi-bin/ComputParamsV2.py

## Abbreviations

AMPs: antimicrobial peptides
CD: circular dichroism
GM: geometric mean
hRBC: human red blood cell
LPS: lipopolysaccharide
MBC: minimum bactericidal concentration
MHC: minimal hemolytic concentration
MICs: minimum inhibitory concentrations
MTT: 3-(4,5-dimethylthiozol-2-yl)-2,5-diphenyltetrazolium bromide
NPN: 1-N-phenylnapthylamine
ONPG: o-nitrophenyl - β -D-galactopyranoside
PI: propidium iodide
POPC: 1-palmitoyl-2-oleoyl-sn-glycero-3-phosphocholine
POPG: 1-palmitoyl-2-oleoyl-sn-glycero-3-phospho-(10-rac-glycerol)
SDS: Sodiumdodecylsulfate
SEM: scanning electron microscopy
SUV: small unilamellar vesicles
TEM: transmission electron microscopy
TFE: trifluoroethyl alcohol
TI: therapeutic index.
